# Accelerated ^99m^Tc-sestamibi clearance associated with mitochondrial dysfunction and regional left ventricular dysfunction in reperfused myocardium in patients with acute coronary syndrome

**DOI:** 10.1186/s13550-016-0196-5

**Published:** 2016-05-12

**Authors:** Atsuro Masuda, Keiichiro Yoshinaga, Masanao Naya, Osamu Manabe, Satoshi Yamada, Hiroyuki Iwano, Tatsuya Okada, Chietsugu Katoh, Yasuchika Takeishi, Hiroyuki Tsutsui, Nagara Tamaki

**Affiliations:** Department of Nuclear Medicine, Hokkaido University Graduate School of Medicine, Sapporo, Japan; Molecular Imaging Research Center, National Institute of Radiological Sciences, 4-9-1 Anagawa, Inage-Ku, Chiba, 263-8555 Japan; Department of Cardiovascular Medicine, Hokkaido University Graduate School of Medicine, Sapporo, Japan; Department of Health Sciences, Hokkaido University Graduate School of Medicine, Sapporo, Japan; Department of Cardiology and Hematology, Fukushima Medical University, Fukushima, Japan; Department of Natural Sciences, Fukushima Medical University, Fukushima, Japan

**Keywords:** Acute coronary syndrome, Clearance, Metabolism, Sestamibi

## Abstract

**Background:**

Accelerated clearance of ^99m^technetium-sestamibi (MIBI) has been observed after reperfusion therapy in patients with acute coronary syndrome (ACS), but the mechanisms have not been fully investigated. MIBI retention may depend on mitochondrial function. The clearance rate of ^11^carbon-acetate reflects such mitochondrial functions as oxidative metabolism. The purpose of this study was to examine the mechanisms of accelerated MIBI clearance in ACS. We therefore compared it to oxidative metabolism estimated using ^11^C-acetate positron emission tomography (PET).

**Methods:**

Eighteen patients [mean age 69.2 ± 8.7 years, 10 males (56 %)] with reperfused ACS underwent MIBI single-photon emission computed tomography (SPECT), echocardiography, and ^11^C-acetate PET within 3 weeks of the onset of ACS. MIBI images were obtained 30 min and 3 h after MIBI administration. Regional left ventricular (LV) function was evaluated by echocardiography. The measurement of oxidative metabolism was obtained through the mono-exponential fitting of the ^11^C-acetate time-activity curve (*k*_mono_).

**Results:**

Among 95 segments of reperfused myocardium, MIBI SPECT showed 64 normal segments (group N), 14 segments with accelerated MIBI clearance (group AC), and 17 segments with fixed defect (group F). Group AC showed lower *k*_mono_ than group N (0.041 ± 0.009 vs 0.049 ± 0.010, *p* = 0.02). Group F showed lower *k*_mono_ than group N (0.039 ± 0.012 vs 0.049 ± 0.010, *p* = 0.01). However, *k*_mono_ was similar in group AC and group F (*p* = 0.99).

**Conclusions:**

Segments with accelerated MIBI clearance showed reduced oxidative metabolism in ACS. Loss of MIBI retention may be associated with mitochondrial dysfunction.

## Background

Acute coronary syndrome (ACS) induces myocardial ischemia followed by myocardial cell injury. ACS-induced myocardial cell injury may also cause mitochondrial dysfunction. Myocardial mitochondrial dysfunction is thought to be associated with the process of myocardial cell death [1]. Early intervention to protect mitochondrial function may also be important for myocyte protection [2]. Therefore, accurate detection of mitochondrial dysfunction in patients with ACS is considered to be important.

^99m^Technetium-sestamibi (MIBI) is a lipophilic and cationic agent that is passively taken up by myocytes after intravenous administration. MIBI is distributed on the mitochondrial membrane in relation to the electrical gradient [3, 4]. Accelerated MIBI clearance has been observed in patients with acute myocardial infarction after reperfusion therapy [5], and it may be a predictor of left ventricular (LV) functional improvement at follow-up [6].

^11^C-acetate positron emission tomography (PET) can non-invasively evaluate myocardial oxidative metabolism [7–12] and myocardial blood flow [13, 14]. ^11^C-acetate clearance is associated with citric acid cycle activity in the mitochondria, in which acetate is converted into acetyl-CoA and metabolized via the action of acetyl-CoA synthetase 2 [15]. Therefore, oxidative metabolism as estimated using ^11^C-acetate PET can be associated with mitochondrial function. Previous studies have suggested an association between accelerated MIBI clearance and mitochondrial dysfunction in dilated and hypertrophic cardiomyopathy based on experimental researches [16]. However, no previous study has looked at the pathophysiological mechanisms of accelerated MIBI clearance in patients with ACS.

The purpose of this study was to examine the mechanism of accelerated MIBI clearance in patients with ACS. Therefore, we compared it to oxidative metabolism estimated using ^11^C-acetate PET. The second aim of this study was to evaluate the association between regional accelerated MIBI clearance and regional LV functional recovery.

## Methods

### Study subjects

ACS patients were prospectively recruited from the Department of Cardiovascular Medicine at the Hokkaido University Hospital from August 2006 to February 2012. We enrolled patients diagnosed with ACS [17] who had revascularization immediately after admission to the hospital [mean age 69.2 ± 8.7 years, 10 males (56 %)]. ACS included unstable angina, non-ST-elevated myocardial infarction (NSTEMI), and ST-elevated myocardial infarction (STEMI) [18]. Exclusion criteria were (1) patients with prior myocardial infarction, (2) patients who were younger than 20 years old, and (3) patients whose condition was unstable. The study was approved by the Hokkaido University Graduate School of Medicine Human Research Ethics Board. Written informed consent was obtained from all patients.

### Study protocol

**Fig. 1:**
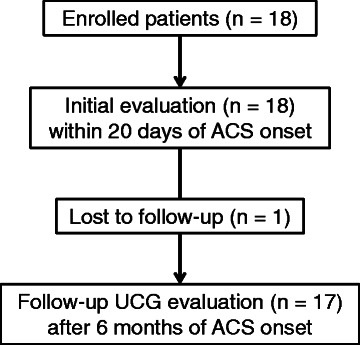
Study protocol. *ACS* acute coronary syndrome

Within 20 days of the onset of ACS, patients had rest and delayed rest MIBI single-photon emission computed tomography (SPECT), rest ^11^C-acetate PET, and echocardiography at rest. These three imaging data acquisitions were performed within 7 days. The interval between nuclear imaging and echocardiography was 1.7 ± 2.4 days (Fig. 1). Follow-up echocardiography was performed 6 months after the onset of ACS (Fig. 1).

### MIBI SPECT myocardial perfusion imaging

MIBI SPECT myocardial perfusion imaging was performed at rest. Six hundred megabecquerels (MBq) of MIBI (FUJIFILM RI Pharma, Tokyo, Japan) was intravenously administered at rest. Standard data acquisition was performed 30 min after MIBI administration [19], and an additional rest SPECT data acquisition was performed 180 min after MIBI administration [5].

All images were obtained using a dual-detector gamma camera (Millennium MG, General Electric, Elgems, Tirat Carmel, Israel) equipped with a parallel hole, low-energy, high-resolution collimator. Energy discrimination was provided by a 20 % window centered at 140 keV. Thirty-two images were obtained over a 180° arc. Each image was acquired over 30 s. The data were stored on a 64 × 64 matrix. A series of 6.78-mm-thick contiguous trans-axial images were reconstructed with a filtered back-projection algorithm without attenuation correction. These trans-axial images were then reoriented in the short axis, vertical long axis, and horizontal long axis of the left ventricle.

### MIBI SPECT image interpretation

The LV wall was divided into 16 segments based on the American Society of Echocardiography (ASE) recommendations [20]. Each basal and mid-ventricular region was divided into six segments, and the apical region was divided into four segments. The border between the anteroseptal and anterior segments was considered to be at the anterior insertion of the right ventricular wall into the left ventricle. Also, the inferior insertion of the right ventricular wall into the left ventricle defined the border between the inferoseptal and inferior segments [20]. The association between the myocardial segments and the three major coronary arteries was also defined based on the ASE recommendations [20]. The left anterior descending artery (LAD) region included six segments, the left circumflex artery (LCx) region included five segments, and the right coronary artery (RCA) region included five segments. Two nuclear cardiologists independently performed visual evaluation of myocardial perfusion imaging. The observers performed image evaluations blinded to the patients’ clinical information and other imaging data. Discordant findings were resolved by a third observer. A standard five-point visual scoring system was used for evaluating regional myocardial MIBI uptake: 0 normal perfusion, 1 mild reduction, 2 moderate reduction, 3 severe reduction, and 4 absent uptake [19, 21]. Accelerated MIBI myocardial clearance was defined as an increase of 1 or more in the segmental defect score at the additional delayed rest image obtained at the 3-h mark after MIBI administration as compared with the early rest image [5]. MIBI redistribution was defined as a decrease of 1 or more in the segmental defect score at the delayed rest image as compared with the early rest image in segments with reduced uptake at initial rest imaging [22]. The percentage peak uptake was also analyzed in each LV segment using the Heart Function View software (Nihon Medi-Physics Co., Ltd., Tokyo, Japan).

### Echocardiography

Echocardiography was performed early after reperfusion therapy and at 6 months after the onset of ACS (Fig. 1). All echocardiographic examinations were performed by experienced echocardiographers blinded to the clinical information, PET image findings, and SPECT image findings. We used a commercially available ultrasound system (Sonos 5500, Philips Medical Systems, Andover, MA, USA) equipped with a broadband harmonic phased-array transducer (S4 probe). LV wall motion was evaluated using the 16-segment model based on the ASE recommendations [20]. LV wall motion was evaluated using a five-point scoring system according to the ASE guidelines: 1 normokinesis, 2 mild hypokinesis, 3 severe hypokinesis, 4 akinesis, and 5 dyskinesis [20]. Six months after the onset of ACS, we evaluated the regional wall motion as a follow-up study (Fig. 1). A decrease of more than 1 in the LV wall motion score at follow-up compared with the initial study was defined as regional wall motion improvement [23]. Left ventricular hypertrophy (LVH) was defined as interventricular septal wall thickness or posterior wall thickness of 11 mm or more [20].

### ^11^C-acetate positron emission tomography

Patients were instructed to fast for at least 6 h prior to ^11^C-acetate PET study. Patients were positioned with the heart centered in the field of view in a whole-body PET scanner (ECAT HR+, Siemens/CTI Knoxville, TN, USA) [9, 24]. A dynamic PET acquisition was initiated (10 × 10, 2 × 30, 5 × 100, 3 × 180, and 2 × 300 s) [10, 25] just after intravenous administration of 740 MBq of ^11^C-acetate. Blood pressure, heart rate, and electrocardiography were monitored during the PET scans.

### ^11^C-acetate PET data analysis

PET image data were analyzed using an image analysis package (Dr. View; Asahi Kasei, Tokyo, Japan) and a special dedicated in-house software called the Hokkaido Quantitative Tool (HOQUTO) [9, 26]. The images were iteratively reconstructed and resliced along the short axis [27]. Based on the ASE recommendations [20], regions of interest were defined for each of the 16 segments.

Regional oxidative metabolism was determined from the mono-exponential function (*k*_mono_) fit to the linear portion of the semilogarithmic plot. The mono-exponential fit began at the point where the blood pool was stable (usually 2 to 4 min after injection) as previously described [8, 24, 28]. All data were analyzed by nuclear cardiologists blinded to clinical information and other imaging data.

### Statistical analysis

Continuous variables were expressed as mean plus standard deviation. Categorical variables were described as number and percentage. The Wilcoxon signed-rank test was performed between the initial and follow-up echocardiographic measurements. We evaluated the difference in oxidative metabolism (*k*_mono_) among the three types of segments, namely those in group N, group AC, and group F, using a linear mixed effects model. Random effects were defined as subject, and fixed effects were defined as segment type. Then, we analyzed the differences among the three groups using multi-group comparison. Logistic regression analysis was performed to analyze the relationship between the time of ACS onset to reperfusion and the presence or absence of accelerated MIBI clearance. All statistical analyses were performed using R version 3.0.2 (The R Foundation for Statistical Computing, Vienna, Austria).

## Results

### Baseline patient characteristics

We enrolled 18 ACS patients who had immediate revascularization for the culprit vessel. No patient had a diagnosis of hypertrophic cardiomyopathy. Nine patients were revascularized for LAD. Two patients were revascularized for the LCx artery and the remaining seven patients for the RCA (Table 1). All patients were revascularized using a coronary artery stent and obtained TIMI flow grade 3 by the end of percutaneous coronary intervention (PCI). The required time from ACS onset to revascularization was 5.4 ± 6.8 h. Peak creatine kinase (CK) level was 1697.6 ± 1522.5 IU/L (range 112–5056 IU/L, Table 2). One patient had already undergone PCI for the LAD artery due to angina pectoris prior to ACS onset. However, this patient’s culprit region at the ACS event was the RCA. Therefore, we included this patient in the present study. Detailed information related to the ACS events of each patient is provided in Table 2.

**Table 1:** Baseline patient characteristics. Data are *n*, with percentages in parentheses, or mean ± SD, unless otherwise indicated

Characteristic	All patients (*n* = 18)
Age, years	69.2 ± 8.7
Male	10 (56 %)
Culprit region	
LAD	9 (50 %)
LCx	2 (11 %)
RCA	7 (39 %)
Time from onset to revascularization, h	5.4 ± 6.8
Revascularization method, *n* (%)	
Stent	18 (100 %)
Peak creatine kinase, IU/L	1697.6 ± 1522.5
Coronary risk factor, *n* (%)	
Hypertension	9 (50 %)
Diabetes mellitus	5 (28 %)
Dyslipidemia	10 (56 %)
Smoking history	9 (50 %)
Past history	
Pacemaker implantation	1 (6 %)
Post PCI	1 (6 %)
Hemodynamics	
Systolic blood pressure, mmHg	122.7 ± 14.9
Diastolic blood pressure, mmHg	62.3 ± 9.9
Heart rate, beats per min	63.3 ± 9.2
Echocardiography data	
LVEF, %	56.5 ± 9.5
Interventricular septal wall thickness, mm (range)	10.4 ± 1.9 (7–16)
Posterior wall thickness, mm (range)	9.2 ± 1.1 (7–12)

*LAD* left anterior descending artery, *LCx* left circumflex artery, *RCA* right coronary artery, *PCI* percutaneous coronary intervention, *LVEF* left ventricular ejection fraction

**Table 2:** Detailed patients’ information about their ACS events

Pt	Culprit vessel	LVH	Peak CK (IU/L)	Time from onset to revascularization (h)	Accelerated MIBI clearance in the culprit region
1	RCA	No	374	2.4	No
2	LCx	Yes	268	NA	No
3	RCA	No	853	5.0	No
4	RCA	No	1550	5.0	Yes
5	LAD	Yes	612	NA	Yes
6	LAD	Yes	4189	5.0	No
7	RCA	No	1943	1.5	Yes
8	LAD	Yes	1133	4.2	No
9	LAD	No	4874	3.0	Yes
10	LAD	Yes	5056	3.0	Yes
11	LAD	Yes	952	NA	Yes
12	LAD	No	969	1.0	No
13	RCA	Yes	2040	4.0	Yes
14	RCA	No	2415	4.5	Yes
15	LCx	Yes	112	NA	No
16	LAD	No	617	3.0	Yes
17	RCA	No	1411	28.5	Yes
18	LAD	No	1188	6.0	No

*Pt* patient, *LVH* left ventricular hypertrophy, *RCA* right coronary artery, *LCx* left circumflex artery, *LAD* left anterior descending artery, *CK* creatine kinase, *MIBI* ^99m^Tc-sestamibi, *NA* not available

### Echocardiography findings

Initial echocardiography study was performed 7.4 ± 2.8 days after ACS onset (range 2–12 days). LVH was observed in eight patients based on the ASE guidelines criteria [20]. At the initial study, group AC (*n* = 14) showed higher wall motion scores than group N (*n* = 64) (2.29 ± 0.99 vs 1.47 ± 0.62, *p* < 0.01). Group F also showed higher wall motion scores than group N (*n* = 17) (2.41 ± 1.06 vs 1.47 ± 0.62, *p* < 0.01).

### MIBI SPECT findings

MIBI SPECT was performed 8.9 ± 3.7 days after ACS onset (range 3–20 days). Accelerated MIBI clearance was observed in 10 out of 18 patients (56 %). The time from ACS onset to revascularization was not associated with the presence or absence of accelerated MIBI clearance (*p* = 0.52). There was no significant correlation between the time from revascularization to SPECT and numbers of segments (*R* = 0.23, *p* = 0.36). In addition, the time from ACS onset to initial MIBI SPECT imaging was not associated with the presence or absence of accelerated MIBI clearance (*p* = 0.20). Among a total 288 LV segments, we evaluated 99 segments related to revascularized coronary arteries. Four segments showed MIBI redistribution in a delayed rest image, and these segments were excluded from the analysis [29]. Among the remaining 95 segments, 64 were defined as having normal myocardial perfusion at early and delayed rest images (group N). Fourteen segments showed accelerated MIBI clearance (group AC), and 17 segments showed fixed perfusion defect (group F).

In group AC, the defect score significantly increased at delayed rest images as compared with early rest images (*p* < 0.001) (Table 3). Group N and group F showed similar defect scores at early and delayed rest images (group N *p* = 0.32 and group F *p* = 0.33) (Table 3). The percentage peak uptake was analyzed in 17 patients. Reduction of percentage peak uptake tended to be higher in group AC than in group N (Tables 4 and 5).

**Table 3:** MIBI SPECT defect score

Segment numbers	Early image	Delayed image	*p* value
Group N (*n* = 64)	0.03 ± 0.12	0.04 ± 0.14	0.32
Group AC (*n* = 14)	0.75 ± 0.87	1.96 ± 0.89	<0.001
Group F (*n* = 17)	2.15 ± 0.77	2.18 ± 0.77	0.33

Data expressed as mean ± SD, unless otherwise indicated

*MIBI* ^99m^Tc-sestamibi, *group N* segments showed normal perfusion in MIBI scintigraphy in rest and delayed images, *group AC* segments showed accelerated MIBI clearance by increase of one or more in defect score in MIBI scintigraphy in delayed image, *group F* segments showed fixed perfusion defect in MIBI scintigraphy in rest and delayed images

**Table 4:** MIBI SPECT percentage peak uptake

Segment numbers	Early image, %	Delayed image, %	*p* value
Group N (*n* = 61)	71.4 ± 13.0	70.2 ± 12.5	0.047
Group AC (*n* = 14)	61.1 ± 12.3	57.9 ± 13.0	0.08
Group F (*n* = 15)	53.7 ± 10.8	48.1 ± 10.7	<0.001

Data expressed as mean ± SD, unless otherwise indicated

*MIBI* ^99m^Tc-sestamibi, *group N* segments showed normal perfusion in MIBI scintigraphy in rest and delayed images, *group AC* segments showed accelerated MIBI clearance by increase of 1 or more in defect score in MIBI scintigraphy in delayed image, *group F* segments showed fixed perfusion defect in MIBI scintigraphy in rest and delayed images

**Table 5:** Percent change of percentage peak uptake

Segment numbers	Percent change of percentage peak uptake
Group N (*n* = 61)	−1.2 ± 5.4
Group AC (*n* = 14)	−3.2 ± 5.9
Group F (*n* = 15)	−5.6 ± 4.2*

*p = 0.025 vs group N

### Regional left ventricular oxidative metabolism

**Fig. 2:**
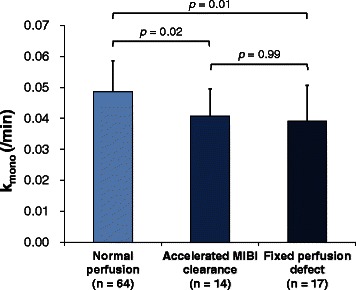
Oxidative metabolism (*k*_mono_) in each segment among the three groups

**Fig. 3:**
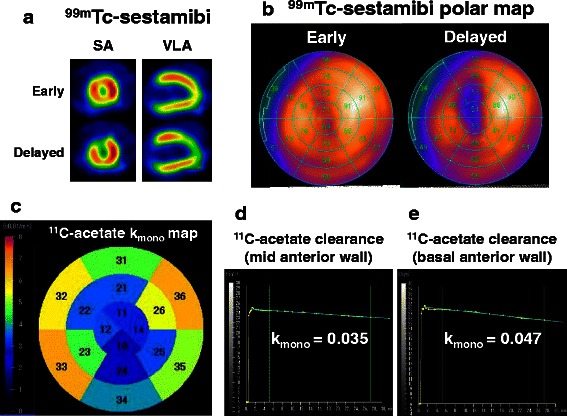
Representative case of an 85-year-old man who underwent emergent PCI for LAD. ^99m^Tc-sestamibi (MIBI) scintigraphy and ^11^C-acetate PET were performed 10 days after PCI. **a** Early and delayed images of MIBI SPECT. Accelerated MIBI clearance is observed in the anterior region. **b** Polar maps of MIBI in early (*upper*) and delayed (*lower*) images. **c** Oxidative metabolism (*k*_mono_) in each segment. **d** ^11^C-acetate PET time-activity curve at the mid-anterior wall. Mid-anterior wall exhibited accelerated MIBI clearance in MIBI scintigraphy. **e** ^11^C-acetate PET time-activity curve at the basal-anterior wall. Basal-anterior wall showed normal perfusion in rest and delayed images in MIBI scintigraphy. *PCI* percutaneous coronary intervention, *PET* positron emission tomography, *LAD* left anterior descending artery, *SA* short axis, *VLA* vertical long axis

^11^C-acetate PET was performed 8.8 ± 2.9 days after ACS onset (range 3–13 days). Group AC showed a lower oxidative metabolism (*k*_mono_) than group N (0.041 ± 0.009 vs 0.049 ± 0.010, *p* = 0.02) (Figs. 2 and 3). Group F also showed a lower oxidative metabolism (*k*_mono_) than group N (0.039 ± 0.012 vs 0.049 ± 0.010, *p* = 0.01). However, there was no difference in oxidative metabolism (*k*_mono_) between group AC and group F (*p* = 0.99).

### Follow-up echocardiography evaluation

**Fig. 4:**
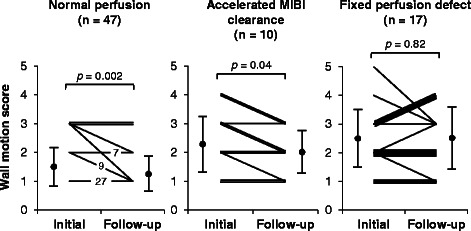
Changes in echocardiographic regional LV wall motion score between initial study and follow-up study

At the follow-up (mean follow-up 7.8 ± 4.5 months), three patients had in-stent restenosis in the reperfused coronary artery region as shown through coronary angiography. One patient did not have follow-up echocardiography. Therefore, these four patients were excluded from the follow-up echocardiographic study, and we evaluated regional wall motion changes between the initial and follow-up study in 14 patients. Among 224 segments in these 14 patients, 74 segments were related to revascularized coronary arteries, and we evaluated these segments as a follow-up study. Group N showed improved LV wall motion score compared with that at its initial study (*n* = 47) (1.47 ± 0.62 to 1.25 ± 0.53, *p* = 0.002) (Fig. 4). Group AC also showed improved LV wall motion score compared with that at its initial study (*n* = 10) (2.29 ± 0.99 to 2.00 ± 0.82, *p* = 0.04). In contrast, the regional wall motion score in group F did not change compared with that at its initial evaluation (*n* = 17) (2.41 ± 1.06 to 2.47 ± 1.12, *p* = 0.82).

## Discussion

Segments with accelerated MIBI clearance were associated with impaired myocardial oxidative metabolism as evaluated by ^11^C-acetate PET in patients with reperfused ACS. Segments with accelerated MIBI clearance also showed impaired regional wall motion. LV wall motion in accelerated MIBI clearance improved at follow-up in patients with reperfused ACS.

### Accelerated MIBI clearance and mitochondrial dysfunction

Approximately 90 % of MIBI accumulates on the mitochondrial membrane in relation to its electrical gradient [3, 30]. In experimental studies, loss of mitochondrial membrane potential was associated with a decrease in MIBI uptake [3]. Myocardial cell injury caused accelerated MIBI clearance in ischemic myocardium in a canine model [31, 32]. In addition, in hypertrophic cardiomyopathy, there was an association between accelerated MIBI clearance and the change of mitochondrial structure as observed in histological examinations [16, 33]. Hayashi et al. reported that accelerated MIBI clearance was correlated with the severity of degeneration in the mitochondria in dilated cardiomyopathy [16]. In the current study, segments with accelerated MIBI clearance showed decreased myocardial oxidative metabolism in patients with ACS. Therefore, reduced oxidative metabolism may reflect mitochondrial dysfunction in ischemic myocardium as well as in either hypertrophic or dilated cardiomyopathy. These data appear to corroborate those from previous experimental studies, and the current data may therefore clarify the possible mechanisms of accelerated MIBI clearance in patients with ACS.

In the current study, accelerated MIBI clearance was observed in ten patients (55 %), and segmental analysis showed that 14 of the 99 segments (14 %) exhibited accelerated MIBI clearance. Takeishi et al. reported that 15 of the 22 patients (68 %) with acute myocardial infarction showed accelerated MIBI clearance [5]. In their study, these patients had percutaneous transmural coronary angioplasty and thrombolytic therapy. The frequency of accelerated MIBI in the current study was lower than in Takeishi’s study. In their study, all patients who underwent angioplasty obtained TIMI grades 2 or 3 by the end of the revascularization procedure. The difference in the frequency of accelerated MIBI between the current study and Takeishi’s study may be due to differences in coronary intervention approach. In the current study, all patients underwent stent placement as the ACS treatment. In addition, all patients had obtained TIMI grade 3 by the end of the revascularization procedure. Given recent developments in treatment approaches, ACS treatments may have improved in comparison with those used in previous studies. Therefore, newer revascularization approaches may result in less myocardial damage and a lower frequency of accelerated MIBI clearance. Even with the lower frequency of accelerated MIBI clearance, the main finding of the current study agreed with the findings from the previous study, and the current study adds new pathophysiological insights over the previous study.

Fujiwara et al. reported that the regions of accelerated MIBI clearance were closely correlated with those showing reduced uptake of ^123^iodine-beta-methyl-iodophenyl-pentadecanoic acid (BMIPP) [34]. BMIPP defect is associated with abnormal myocardial fatty acid metabolism [35]. Their data indicate that accelerated MIBI clearance may be associated with myocardial metabolic dysfunction in ACS. Although they did not evaluate oxidative metabolism, their data may support the current findings.

In the current study, reduction of percentage peak uptake in the segments of accelerated sestamibi clearance showed a trend of being higher than that in normal perfusion segments. However, this was not significant. Since tracer decay might have impacts on the percentage peak uptake, this may have had an influence on the relative uptake analysis.

### Accelerated MIBI clearance and wall motion recovery

The regions with accelerated MIBI clearance showed LV wall motion improvement at follow-up after revascularization. Using low-dose dobutamine stress echocardiography, Fujiwara et al. evaluated the association between accelerated MIBI clearance and regional wall motion after ACS (within 7 days of admission) [6]. Segments with accelerated MIBI clearance showed better functional recovery during low-dose dobutamine administration than those with fixed MIBI defects. Their study revealed accelerated MIBI clearance associated with dysfunctional but viable myocardium early after ACS. The current study further added to the new insight that accelerated MIBI clearance in segments was related to regional wall motion recovery at follow-up. Thus, the current study provided insights into the association between accelerated MIBI clearance and regional LV functional recovery in addition to those provided by previous studies [5, 6].

Myocardial oxidative metabolism in the segments with accelerated MIBI clearance decreased as the number of segments with fixed perfusion abnormality did. Segments with accelerated MIBI clearance showed improved LV wall motion. However, the LV wall motion in segments with fixed perfusion abnormality remained unchanged at follow-up. Thus, based on the current findings, it may be difficult to predict regional wall motion recovery using ^11^C-acetate PET data. The effectiveness of ^11^C-acetate PET to predict myocardial functional recovery has not been fully recognized [36–40]. Hicks et al. reported that oxidative metabolism did not depend on myocardial perfusion. They also reported that oxidative metabolism varied in accordance with myocardial conditions such as ischemia or infarction [37]. They concluded that predicting functional recovery through the evaluation of oxidative metabolism alone was difficult. Our results suggest that segments with impaired oxidative metabolism were associated with myocardial injury. However, this finding was not sufficient to predict myocardial functional recovery based on the current data. Therefore, further studies are required.

### Clinical indication

Evaluation of accelerated MIBI clearance may provide additional information in a clinical setting. Our study may indicate that segments with accelerated MIBI clearance represent myocardium damage as a result of mitochondrial dysfunction.

### Limitations

Our study had some limitations. First, histological findings for the regions showing accelerated MIBI clearance were not evaluated. Rather than performing biopsy sampling, we evaluated myocardial oxidative metabolism as a marker of mitochondrial dysfunction using ^11^C-acetate PET. In experimental studies, the clearance of ^11^C-acetate from myocardium is associated with mitochondrial function [15]. Therefore, the current data support our hypothesis. Second, myocardial oxidative metabolism was not evaluated at follow-up. A previous study reported an improvement in oxidative metabolism in the reperfused myocardium [37]. Examining this information with regard to physiological changes to ACS after reperfusion therapy might be an important next step. Third, for evaluation purposes, we did not separate patients with ACS into groups based on the following pathological conditions: unstable angina, NSTEMI, and STEMI. These three types of pathological conditions may exhibit related pathology in terms of myocardial damage. Finally, our study involved a small study population. The current protocol included two sestamibi SPECT data acquisitions, echocardiography, and one ^11^C-acetate PET study. ^11^C-acetate PET is usually applied for specific pathophysiological studies in a limited number of research facilities [25, 41], and it would be difficult to apply this comprehensive study protocol to a large number of subjects. In addition, the sample size of the present study was small but similar to that of previous studies [39, 41, 42]. Despite the small sample size, with careful preparation, we showed the pathophysiological mechanism of accelerated sestamibi washout. While a small sample size may have had a minimal impact on the current data, we definitely need further study using a larger study population to confirm the efficacy of evaluating MIBI clearance. The time from revascularization to SPECT showed some variability. However, there was no significant correlation between the time from revascularization to SPECT and the numbers of accelerated sestamibi clearance segments. Therefore, time from revascularization to SPECT might not have had an impact on the numbers of segments with accelerated sestamibi clearance.

## Conclusions

Segments with accelerated MIBI clearance were associated with impaired myocardial oxidative metabolism as evaluated by ^11^C-acetate PET. Segments with accelerated MIBI clearance showed impaired regional wall motion. Accelerated MIBI clearance may be associated with mitochondrial dysfunction and might be a predictor of LV wall motion improvement in patients with ACS who underwent immediate revascularization therapy.

## Abbreviations

ACS: acute coronary syndrome
LAD: left anterior descending artery
LCx: left circumflex artery
LV: left ventricular
MIBI: ^99m^technetium-sestamibi
PCI: percutaneous coronary intervention
PET: positron emission tomography
RCA: right coronary artery
SPECT: single-photon emission computed tomography
